# Supplementary material for: Accelerated corrosion of low carbon steel by oscillatory acidic streams generated with a bio-inspired claw device
Source: PLoS One. 2024 Apr 4;19(4):e0298266. doi: 10.1371/journal.pone.0298266 (PMC10994280; doi:10.1371/journal.pone.0298266)
Supplement: S1 Appendix — (PDF) [file pone.0298266.s001.pdf]

Supporting information

S1 Appendix Calculation of flow rate.
The velocity, $v(t)$, of the flow was estimated according to the following equation:

$$v(t) = \frac{\dot{V}(t)}{A(t)}$$

Where $\dot{V}(t)$ represents the volumetric flow generated by the cyclic motion of the movable claw, while $A(t)$ represents the area of the mouthpiece as a function of time, through which fluid enters the socket or exits from inside the socket. S1A Fig b shows two positions (at different instants) of the mobile claw, where the mouthpiece area A, which changes as a function of θ and time, is plotted. To estimate $A(t)$, we first measured the shaded area (indicated in blue, see S1A Fig b) corresponding to the mouthpiece (using ImageJ software [46]) for different values of theta in the range $5.4° \leq \theta \leq 34.3°$, whose limits correspond to the total closing and opening positions of the mobile claw, and thus obtained an estimate of the function $A(\theta)$. But the angular displacement θ is a function of time (as shown in Figure 2a), and so in direct form is how the curve of $A(t)$ shown in S1A Fig a was obtained. To estimate$V(t)$, the lateral area of socket $A1$ was measured (see S1A Fig d) for different values of theta in the range $5.4° \leq \theta \leq 34.3°$; these data were multiplied by the thickness "ϵ" of the plate with which the claw is constructed, and thus an estimate of the volume of the socket was obtained as a function of the angle θ, which as we know is in turn a function of time, and thus in direct form is how the curve of $V(t)$ shown in S1A Fig c was obtained. Finally, $\dot{V}(t)$, was obtained by numerically differentiating the function $V(t)$.

S1A Fig. a) Mouthpiece area vs. time. b) Different opening positions of the mobile claw to show how the surface A (represented by the blue surface) varies as a function of the angular position and therefore of time. In position (1) the claw is in its initial condition with the smallest mouthpiece area, in position (2) the maximum opening is shown and consequently also A is maximum. c) Volume of the socket formed by the internal side walls of the two support plates (6), as shown in Figure 1, the internal side walls of both the fixed and mobile claws and of course the area A of the mouthpiece. d) Two opening positions of the mobile claw to indicate the area $A1$ (shown in purple color), as well as the internal volume of the socket, the latter corresponding to the intermediate position (3). Clearly, the volume of the socket changes with the theta angle and also with time. The symbols on the $A(t)$ and $V(t)$ curves correspond to data obtained from area and volume measurements at different angular positions, respectively; the solid lines in both plots represent interpolations using cubic splines to smooth their appearance.
