# Supplementary material for: Accelerated corrosion of low carbon steel by oscillatory acidic streams generated with a bio-inspired claw device
Source: PLoS One. 2024 Apr 4;19(4):e0298266. doi: 10.1371/journal.pone.0298266 (PMC10994280; doi:10.1371/journal.pone.0298266)
Supplement: S2 Appendix — (PDF) [file pone.0298266.s002.pdf]

Supporting information

S2 Appendix Circuit to control the duty cycles of the motor-laser-lamp system.

S2A Fig is a photo of the connections and layout of the electronic components of the circuit to control the on/off of the lamp, laser and motor. Relay (a) was used for motor control while relay (c) was employed for lamp control. Additionally, relay (b) was used to control the laser, which was powered by a DC-DC boost converter MT3608. Each relay is a module with a single channel board that requires 5V DC input to operate. The Arduino board was programmed to control the sequential on/off of the lamp, laser and motor according to different operation patterns.

S2A Fig. Connections and components of the circuit used to control the switching on and off of the devices. Relays to control the a) motor, b) laser , and c) lamp. d) Arduino UNO microcontroller, e) 532 nm (50 mW) laser, f) DC-DC boost converter to power the laser, g) 3mm diameter PMMA fiber optic cable.
