# Supplementary figures and images for: Accelerated corrosion of low carbon steel by oscillatory acidic streams generated with a bio-inspired claw device
Source: PLoS One. 2024 Apr 4;19(4):e0298266. doi: 10.1371/journal.pone.0298266 (PMC10994280; doi:10.1371/journal.pone.0298266)

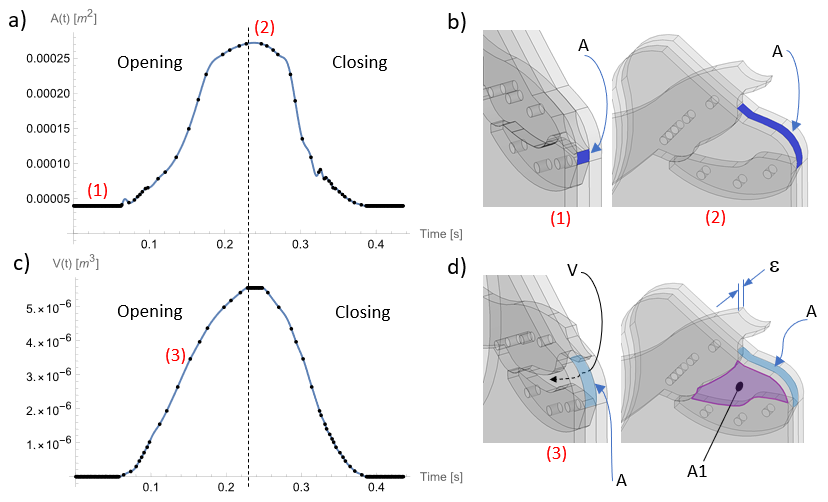

Supplement: S1 Fig — a) Mouthpiece area vs. time. b) Different opening positions of the mobile claw to show how the surface A (represented by the blue surface) varies as a function of the angular position and therefore of time. In position (1) the claw is in its initial condition with the smallest mouthpiece area, in position (2) the maximum opening is shown and consequently also A is maximum. c) Volume of the socket formed by the internal side walls of the two support plates (6), as shown in Fig 1, the internal side walls of both the fixed and mobile claws and of course the area A of the mouthpiece. d) Two opening positions of the mobile claw to indicate the area A1 (shown in purple color), as well as the internal volume of the socket, the latter corresponding to the intermediate position (3). Clearly, the volume of the socket changes with the theta angle and also with time. The symbols on the A(t) and V(t) curves correspond to data obtained from area and volume measurements at different angular positions, respectively; the solid lines in both plots represent interpolations using cubic splines to smooth their appearance. (TIF) [file pone.0298266.s003.tif]

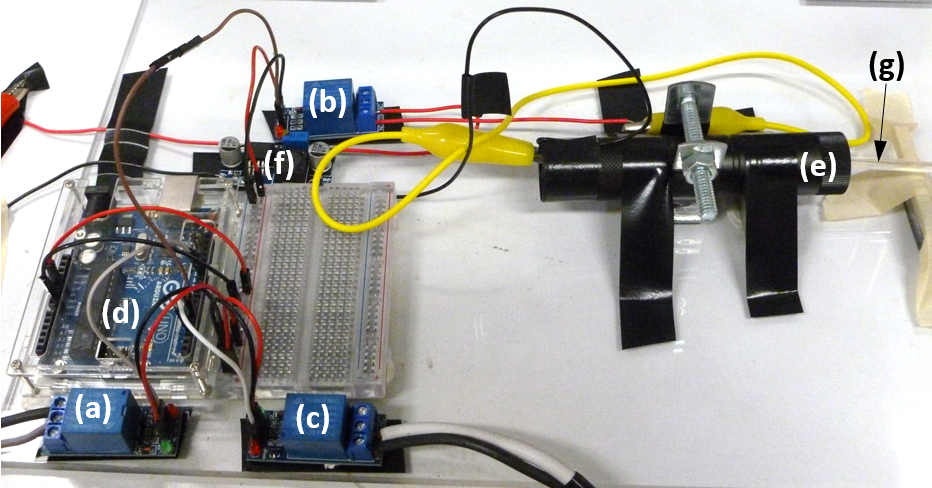

Supplement: S2 Fig — Relays to control the a) motor, b) laser, and c) lamp. d) Arduino UNO microcontroller, e) 532 nm (50 mW) laser, f) DC-DC boost converter to power the laser, g) 3mm diameter PMMA fiber optic cable. (TIF) [file pone.0298266.s004.tif]
